# Hyperkalemia Among Hospitalized Patients and Association Between Duration of Hyperkalemia and Outcomes

**DOI:** 10.7759/cureus.10401

**Published:** 2020-09-12

**Authors:** Tahani Nasser Alrashidi, Razan Ahmed Alregaibah, Khalaf Abdullah Alshamrani, Ahmed A Alhammad, Razan Hamad A Alyami, Mawadda Abdullah Almadhi, Mohamed E Ahmed, Hind Almodaimegh

**Affiliations:** 1 General Physician, College of Medicine, University of Tabuk, Tabuk, SAU; 2 Pharmacology and Therapeutics, College of Pharmacy, Qassim University, Buraidah, SAU; 3 Internal Medicine, College of Medicine, University of Bisha, Bisha, SAU; 4 Emergency Medicine, College of Medicine, King Abdulaziz University, Jeddah, SAU; 5 Internal Medicine, College of Medicine, King Khalid University, Abha, SAU; 6 Emergency Medicine, College of Medicine, King Saud bin Abdulaziz University for Health Sciences, Riyadh, SAU; 7 Biostatistics, King Abdullah International Medical Research Center, Jeddah, SAU; 8 Pharmacology and Therapeutics, College of Pharmacy, King Saud bin Abdulaziz University for Health Sciences and King Abdullah International Medical Research Center, Riyadh, SAU

**Keywords:** hyperkalemia, potassium, adults, drug induced hyperkalemia, acute kidney injury, azole antifungal

## Abstract

Background

Hyperkalemia is a serious life-threatening condition that leads to significant morbidity and mortality.

Objectives

The aim of this study is to investigate the association between the duration and outcomes in patients hospitalized with hyperkalemia, as well as associated risk factors and drug-induced hyperkalemia.

Methods

A three-year retrospective chart review study was conducted at a tertiary hospital at King Abdulaziz Medical City, Riyadh, Saudi Arabia, between January 2016 and April 2019. We included all hospitalized adults and patients with hyperkalemia. Pediatric patients and dialysis patients with end-stage renal failure were excluded.

Results

Out of a total of 300 hospitalized patients who were screened for hyperkalemia, only 142 patients were included. The majority of cases were males (56.3%), whereas 43.7% were females. Most patients were above 55 years old. Regarding comorbidities in patients with hyperkalemia, most of them suffered from high blood pressure or diabetes. The mean serum potassium value was 5.7 ± 0.51 mEq. The most frequent medications used in the study population were azole antifungal medication followed by nonsteroidal anti-inflammatory drugs, beta-blockers, and angiotensin-converting enzyme inhibitors. Around 54 patients were not treated with medication and were monitored for spontaneous correction of hyperkalemia. Insulin was the most used medication for the treatment of hyperkalemia. The mean duration for the resolution of hyperkalemia was 12 ±9.4 hours. Out of 142 patients, only 10 (7%) patients died with hyperkalemia.

Conclusions

Hospitalized patients are at risk of hyperkalemia. In our study, we found that patients who had hyperkalemia were significantly likely to have acute kidney injury or cardiovascular diseases, and azole antifungals and beta-blockers were the most commonly used medications.

## Introduction

Serum potassium plays multiple critical roles and is essential for normal cardiac, nervous, and muscular functions. Several homeostatic factors keep serum potassium within a narrow range, such as insulin, aldosterone, β-adrenergic receptors, and blood pH. Any irregularity in these factors can result in hypo- or hyperkalemia [1]. Hyperkalemia is defined as a high serum level of K+. There is no universal cut-off point for hyperkalemia, but a level of ≥5.5 mEq/L is widely used [2]. Most frequently, hyperkalemia is associated with life-threatening cardiac dysrhythmias and arrest, which significantly increase morbidity and all-cause mortality [3-5]. The time until the resolution of hyperkalemia has been found to be significantly associated with in-hospital mortality, with an incidence of 1.1 to 10 patients per 100 admitted patients [5-6]. Chronic kidney disease, metabolic acidosis, and drugs are considered as the most common risk factors for hyperkalemia [3,5]. Up to 75% of admitted patients have drug-induced hyperkalemia, particularly those using renin-angiotensin-aldosterone system inhibitors (RAASis) [5]. Other conditions and risk factors are hypertension, high protein intake, diabetes mellitus, renal insufficiency, coronary artery disease, and peripheral vascular disease [4,7]. Hyperkalemia is rarely detected in the general population and has a reported incidence of less than 5% worldwide. Studies suggest that the occurrence of hyperkalemia appears to be significant among hospitalized patients [8]. The management of severe hyperkalemia requires both urgent measures and awareness of the underlying cause in order to save lives and achieve maintenance therapy to keep potassium levels within their normal range [9]. However, based on our findings, there are no available data on hyperkalemia among hospitalized Saudi populations. Hence, the aim of this study is to investigate the association between the duration of hyperkalemia and the outcome among hospitalized patients. The results could help the high-risk patient populations that are prevalent in Saudi Arabia.

## Materials and methods

This retrospective chart review study examined well-defined clear questions based on the medical record numbers (MRNs) of patients who attended King Abdulaziz Medical City (KAMC), Riyadh, Saudi Arabia, between January 2016 and April 2019. KAMC is a tertiary hospital and considered one of the major divisions of the National Guard Health Affairs. It was established in May 1983 and has a capacity of 1,505 beds. The hospital provides all types of health services to all National Guard soldiers and their families. The study included all hospitalized patients with a diagnosis of hyperkalemia at the time of admission or during hospitalization who were older than 18 years and had serum potassium ≥5.1 mEq/L. Patients younger than 18 years, dialysis patients with end-stage renal failure, and patients with incomplete data were excluded. Medical records of 300 hospitalized patients were screened for hyperkalemia, of which 142 patients were included. Hyperkalemia was considered as a condition in which the potassium level in the blood is above normal [1]. We obtained approval from the Institutional Review Board of KAIMARC. All patients’ information was kept private and confidential by using coding numbers for each patient’s information. Furthermore, the information was viewed and accessed by only the research members. Patient data were obtained from medical records through their MRNs. We used descriptive questions to examine the following variables: gender, mean age, measured potassium level, number of admissions and reasons, associated medical conditions, medications associated with hyperkalemia, duration of hyperkalemia until resolution, mortality, associated medical complications, and treatment used for hyperkalemia. Data were saved in an appropriately designed Excel spreadsheet.

Statistical analysis

Raw data were processed to identify any inaccuracies or incompleteness before the statistical analysis. To accomplish this, all variables were checked and summarized in terms of maximum and minimum values. The ranges were checked and compared against the possible ranges of each variable, and those with implausible values were flagged. A similar process was applied to categorical variables to identify any potential anomalies, such as miscodes by running a general frequency analysis. The data management and analyses were carried out using the Statistical Package for Social Sciences (SPSS) version 21.0 (IBM Corp., Armonk, NY, USA). Descriptive statistical analyses were carried out, and the results are reported as numbers and percentages for categorical variables and as the mean and standard deviation (SD) for continuous ones. Continuous variables are summarized as the mean ± SD or the median (range). Proportions were used for categorical variables. Demographic and clinical information are summarized in frequency tables. A chi-squared test was used to compare between groups. A multivariate logistic regression analysis was also carried out using the variables that showed statistical significance at the bivariate level. Interaction between these variables was also assessed. The adjusted odds ratio (OR) and 95% confidence interval (CI) are also reported, and the level of significance was determined using p < 0.05.

## Results

A total of 300 hospitalized patients were screened for hyperkalemia, of whom 142 patients were included. The majority of cases were males (56.3%) and 43.7% were females. We divided the patients into three age groups, and most patients (94.4%) were above 55 years old. Regarding comorbidities among hyperkalemia patients, 114 of them suffered from high blood pressure (80.2%), 105 (73.9%) had diabetes, 80 (56.3%) had cardiovascular diseases, 65 (45.8%) had chronic kidney disease, 28 (19.8%) had liver disease, and 25 (17.6%) had tumors (Table 1).

**Table 1:** Baseline characteristics of 142 patients hospitalized with hyperkalemia.

Variable	Result
Age, mean ± standard deviation (years)	56 ± 11
Male %	56.3
Female %	43.7
Hypertension %	80.2
Diabetes mellitus%	73.9
Cardiovascular diseases %	56.3
Acute kidney injury %	45.8
Liver cirrhosis %	19.8
Solid tumors %	17.6
Organ transplantation %	7.7
Tissue necrosis %	4.9
Metabolic acidosis %	3.5
Blood transfusion %	1.4
Dehydration %	0.7

**Figure 1:**
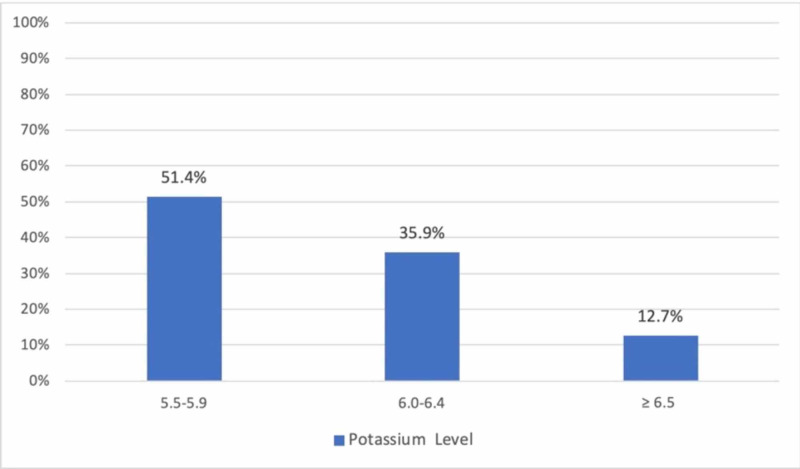
Potassium levels among hospitalized patients.

The mean serum potassium value was 5.7 mEq, and the highest and lowest potassium levels were 6.5 mEq and 5.5 mEq, respectively (Figure 1).

Almost half (47.2%) of the study population were found to use azole antifungal medications, whereas 32.4% used nonsteroidal anti-inflammatory drugs, 31% used beta-blockers, and 30.3% used angiotensin-converting enzyme inhibitors (ACEIs). Penicillin and saline showed similar results (0.7%; Table 2).

**Table 2:** Prevalence of medications associated with hyperkalemia among hospitalized patients.

Medication		Result
Azole antifungals, n (%)		67 (47.2)
Non-steroidal anti-inflammatory drugs, n (%)		46 (32.4)
β-blockers, n (%)		44 (31)
Angiotensin-converting enzyme inhibitors/angiotensin receptor blockers, n (%)		43 (30.3)
Spironolactone/eplerenone, n (%)		17 (12)
Heparin, n (%)		4 (2.8)
Digoxin, n (%)		2 (1.4)
Penicillin, n (%)		1 (0.7)
Saline, n (%)		1 (0.7)

**Figure 2:**
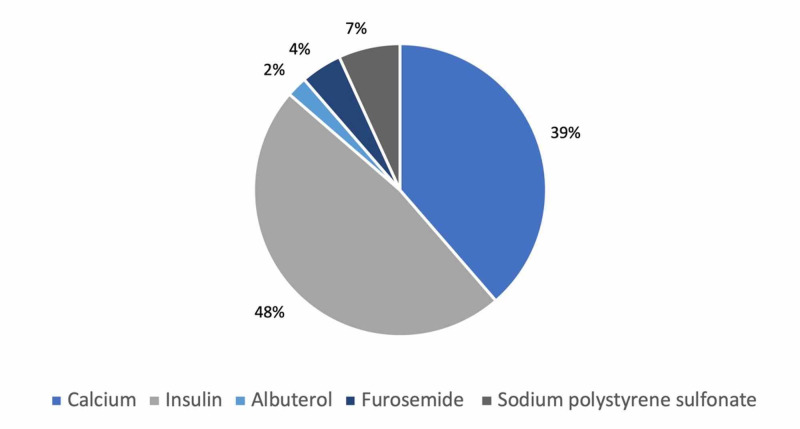
Medications used for the treatment of hyperkalemia.

Intravenous insulin was the most used medication for the treatment of hyperkalemia in 42 (48%) hospitalized patients, whereas 34 (39%) were treated with sodium polystyrene sulfonate. About 54 (38%) patients were not treated with medication and were monitored for spontaneous correction of hyperkalemia (Figure 2).

A total of 81 (57%) patients were admitted for a few days, and a few (19%) were admitted for more than a month. The mean duration for the resolution of hyperkalemia was 12 ± 9.4 hours. Out of 142 patients with hyperkalemia, only 10 (7%) patients died with hyperkalemia.

Hyperkalemia patients were significantly likely to have acute kidney injury and cardiovascular diseases, and azole antifungals and beta-blockers were the most commonly used medications. All other diseases and medications were not significantly correlated with hyperkalemia (Table 3).

**Table 3:** Correlation of hyperkalemia- and hyperkalemia-induced risk factors. ***Indicates significant correlation with p-value < 0.05 ACEI/ARBS, angiotensin-converting enzyme inhibitors/angiotensin receptor blockers; NSAIDs, nonsteroidal anti-inflammatory drugs

Risk factor	p-Value
Acute kidney injury	0.00***
Metabolic acidosis	0.978
Cardiovascular disease	0.012***
Diabetes mellitus	0.152
Hypertension	0.144
Beta-blockers	0.028***
Azole antifungal	0.014***
ACEI/ARBS	0.147
NSAIDs	0.385
Spironolactone/eplerenone	0.226

## Discussion

Hyperkalemia is a common clinical and potentially life-threatening metabolic problem, and its prevalence among Saudi populations is considered high, especially for those with more comorbidities [5]. Our study provides an overall assessment of the rates of potassium testing, patterns, and risk factors of hyperkalemia, as well as the mortality associated with hyperkalemia. In this study, the recorded incidence of hyperkalemia among hospitalized patients who were not on dialysis was 3%, which is similar to the occurrences recorded in preceding surveys [5,10]. Some underlying conditions are associated with the onset of hyperkalemia, such as severe kidney damage, hypertension, congestive heart disorders, and diabetes mellitus. Research also indicates a relationship of tissue necrosis, metabolic acidosis, and blood transfusion with the development of hyperkalemia [11]. The incidence of the disease in our facility was slightly higher than that in other ones [5-6,10]. It could be explained by the mixed varieties of hospitalized patients in our facility, which is one of the largest in the region.

Exposure to different medications is a significant contributor to hyperkalemia [6]. Of the 300 patients involved in the study, 75% indicated being on at least one drug that contributes to the onset of hyperkalemia. The high prevalence of hyperkalemia among inpatients is due to the application of some medications, including β-blockers, heparin, and potassium supplements. Other drugs associated with hyperkalemia include aldosterone antagonists and angiotensin receptor blockers [12]. These drugs are used to treat conditions such as myocardial infarction, hypertension, and heart failure [13]. These medications reveal a connection with hyperkalemia, which is consistent with other studies that included the use of β-blockers for patient care [14]. 

The systematic analysis manifests that patients with nonsteroidal anti-inflammatory drug (NSAID)-induced hyperkalemia achieved rapid restoration to a normokalemic state among hospitalized patients. The use of nonsteroidal anti-inflammatory medication results in limited potassium homeostasis by creating relative hyporeninemic hyperaldosteronism, which inhibits the synthesis of renal prostaglandin [15]. The early hyperkalemia resolution is attributed to fast-acting helpful mediations administered using intravenous solutions and the control of NSAIDs. The prevalence of NSAIDs as a cause of hyperkalemia reflects the population, which has a high incidence of kidney diseases and coronary artery diseases, which are less likely to be treated NSAIDs [16]. The method of collecting data to measure exposure to drugs through the records of the drugs administered before a hyperkalemia episode is controversial [17]. The flaw of this method is that it does not capture over-the-counter drugs. Thus, the validity of this information can often be disregarded. However, this information can be applied for cases in which certain drugs are regularly administered to manage chronic conditions. Metabolic acidosis, acute kidney damage, and tissue necrosis are some conditions considered as independent indicators for extended periods before restoration to a state of normokalemia because the severe metabolic imbalances take relatively longer to repair [3].

Hyperkalemia is a condition related to severe medical consequences and higher in-facility transience. The rate of mortality from hyperkalemia tends to increase with the severity of hyperkalemia [18]. However, the advancement from gentle to toxic arrhythmias is irregular [19]. According to McMahon et al., hyperkalemia can become fatal if left unattended, resulting in severe cardiac arrhythmias because of the rapid increase of serum potassium [20]. Another outcome of hyperkalemia is the reduction of latent membrane budding of the myocardium. This situation leads to a diminished myocardial cell conduction rate and a rise in the repolarization rate, which is likely to cause hyperkalemia-induced arrhythmic death [21]. 

It is clear that tissue necrosis, the use of calcium gluconate, metabolic acidosis, potassium supplements, severe kidney damage, and the interval before hyperkalemia firmly reveal a profound association with in-hospital mortality [22]. Metabolic acidosis, necrosis, and kidney injury are factors that lead to a longer duration of hyperkalemia. The prolonged hyperkalemia periods contribute to the association with rising mortality [23]. This report's findings are consistent with those of other studies [22-23].

This study reveals that the use of calcium gluconate in the management of hyperkalemia is associated with increased in-facility mortality [24]. The increase in mortality can be explained in the context of guidelines that stipulate the application of calcium salts in the event of electrocardiographic alterations in patients that indicate severe hyperkalemia-connected mortality among critically sick patients [25]. Furthermore, the duration before a hyperkalemia episode is linked to a high in-hospital mortality rate [25].

Procedural reviews highlight the limited information available to ascertain the most suitable intervention strategy for the management of hyperkalemia. Other studies on the subject of hyperkalemia indicate that little attention has been given to finding the appropriate frequency and period of observing clients with hyperkalemia [26]. Existing frameworks stipulate that after initial actions for hyperkalemia, physicians should recheck the serum potassium within one to two hours [27]. After the initial observation, the frequency of subsequent monitoring can be adjusted depending on the potassium level in the serum. Furthermore, the potential reversibility of the underlying cause plays a significant role in determining the frequency of subsequent monitoring [28]. Since our study indicates an independent association between prolonged duration and adverse outcomes, we conclude that evidence-based intervention for hyperkalemia among inpatients is vital. However, additional research is necessary to determine the value of initiating treatment for hyperkalemia in enhancing the outcomes, particularly concerning in-hospital mortality.

A significant constraint of this investigation is that it used a retrospective observational approach. This approach hinders the extraction of electrocardiograms from all episodes of hyperkalemia. Patient identification was made using ICD-9 (International Classification of Diseases, 9th Revision) diagnosis codes, which made it easy to trace all hyperkalemia cases during the study period. Because chronic kidney disease is a significant contributor, the ability to quantify the frequency of chronic kidney disease using a deliberate glomerular filtration rate is a chief strength of this study [29].

Independent indicators of in-facility mortality of patients with hyperkalemia include prolonged hyperkalemia durations and acute kidney diseases. These conditions are attributed to potassium supplementation, metabolic acidosis, tissue necrosis, and potassium supplementation, as well as the administration of calcium gluconate in the intervention for hyperkalemia. Thus, it is imperative to provide aggressive intervention measures for hyperkalemia for these patients. This study was subject to some limitations being a retrospective study and a single-center study. We recommend the conduction of future studies with a prospective cohort design to identify potential risk factors.

## Conclusions

Hospitalized patients are at risk for hyperkalemia, which is a life-threatening condition associated with significant morbidity and mortality. In this study, the mean duration for the resolution of hyperkalemia was 12 ± 9.4 hours. The most frequent comorbidities observed in patients with hyperkalemia were hypertension followed by diabetes mellitus and cardiovascular diseases. Patients who had hyperkalemia were significantly likely to have acute kidney injury or cardiovascular diseases, and azole antifungals and beta-blockers were the most commonly associated medications. These findings emphasize the importance of recognizing the underlying cause, urgent measures to correct potassium levels, and early management in order to save the lives of patients, as well as achieving maintenance therapy to keep potassium levels within its normal range.

## References

[1] Hall JE, Guyton AC (2016). Guyton and Hall Textbook of Medical Physiology. https://www.elsevier.com/books/guyton-and-hall-textbook-of-medical-physiology/hall/978-1-4557-7005-2.

[2] Alfonzo A, Soar J, MacTier R, Baines R, Chu A, Mann S, MacRury M (2014). Clinical Practice Guidelines: treatment of acute hyperkalemia in adults UK. Renal Assoc.

[3] An JN, Lee JP, Jeon HJ (2012). Severe hyperkalemia requiring hospitalization: predictors of mortality. Crit Care.

[4] Jain N, Kotla S, Little BB (2012). Predictors of hyperkalemia and death in patients with cardiac and renal disease. Am J Cardiol.

[5] Khanagavi J, Gupta T, Aronow WS (2014). Hyperkalemia among hospitalized patients and association between duration of hyperkalemia and outcomes. Arch Med Sci.

[6] Acker CG, Johnson JP, Palevsky PM, Greenberg A (1998). Hyperkalemia in hospitalized patients: causes, adequacy of treatment, and results of an attempt to improve physician compliance with published therapy guidelines. Arch Intern Med.

[7] Kovesdy CP (2011). Epidemiology of hyperkalemia: an update. Kidney Int Suppl.

[8] Simon LV, Hashmi MF, Farrell MW (2020). Hyperkalemia. StatPearls [Internet].

[9] Montford JR, Linas S (2017). How dangerous is hyperkalemia?. J Am Soc Nephrol.

[10] Stevens MS, Dunlay RW (2000). Hyperkalemia in hospitalized patients. Int Urol Nephrol.

[11] Perazella MA (2000). Drug-induced hyperkalemia: old culprits and new offenders. Am J Med.

[12] Schaefer JA, Gales MA (2016). Potassium-binding agents to facilitate renin-angiotensin-aldosterone system inhibitor therapy. Ann Pharmacother.

[13] Pham TT, Miller MJ, Harrison DL, Lloyd AE, Crosby KM, Johnson JL (2012). Cardiovascular disease and non-steroidal anti-inflammatory drug prescribing in the midst of evolving guidelines. J Eval Clin Pract.

[14] Antrobus J, Doolan L, Bethune D (1993). Hyperkalemia and myocardial atonia following cardioselective β-blockade. J Cardiothorac Vasc Anesth.

[15] Plantinga L, Grubbs V, Sarkar U (2011). Nonsteroidal anti-inflammatory drug use among persons with chronic kidney disease in the United States. Ann Fam Med.

[16] Evans M, Palaka E, Furuland H (2019). The value of maintaining normpkalaemia and enabling RAASi therapy in chronic kidney disease. BMC Nephrol.

[17] Reddi AS (2017). Disorders of potassium. Hypokalemia. Fluid, Electrolyte and Acid-Base Disorders.

[18] Rafique Z, Chouihed T, Mebazaa A, Peacock WF (2019). Current treatment and unmet needs of hyperkalaemia in the emergency department. Eur Heart J Suppl.

[19] Indermitte J, Burkolter S, Drewe J, Krähenbühl S, Hersberger KE (2007). Risk factors associated with a high velocity of the development of Hyperkalaemia in hospitalised patients. Drug Safety.

[20] McMahon G, Mendu ML, Gibbons FK, Christopher KB (2012). Association between hyperkalemia at critical care initiation and mortality. Intensive Care Med.

[21] Vincent JL, Abraham E, Kochanek P, Moore F, Mitchell F (2011). Hyperkalemia and hypokalemia. Textbook of Critical Care. 6th ed.

[22] Grodzinsky A, Goyal A, Gosch K (2016). Prevalence and prognosis of hyperkalemia in patients with acute myocardial infarction. Am J Med.

[23] Long B, Warix J, Koyfman A (2019). Hyperkalemia in the emergency department: yes, a need for further evidence, but do not discount what we have. J Emerg Med.

[24] Norring-Agerskov D, Madsen CM, Abrahamsen B (2017). Hyperkalemia is associated with increased 30-day mortality in hip fracture patients. Calcif Tissue Int.

[25] Palmer BF, Clegg DJ (2018). Hyperkalemia across the continuum of kidney function. Clin J Ame Soc Nephrol.

[26] Liu M, Rafique Z (2019). Acute management of hyperkalemia. Curr Heart Fail Rep.

[27] Kovesdy CP (2017). Updates in hyperkalemia: outcomes and therapeutic strategies. Rev Endocr Metab Disord.

[28] Wanner C, Herzog CA, Turakhia MP, Conference Steering Committee (2018). Chronic kidney disease and arrhythmias: highlights from a Kidney Disease: Improving Global Outcomes (KDIGO) Controversies Conference. Kidney Int.

[29] Bielecka-Dabrowa A, Rysz J, Mikhailidis DP, Banach M (2011). What is the risk of hyperkalaemia in heart failure?. Expert Opin Pharmacothe.

